# Reward learning as a potential target for pharmacological augmentation of cognitive remediation for schizophrenia: a roadmap for preclinical development

**DOI:** 10.3389/fnins.2013.00103

**Published:** 2013-06-18

**Authors:** Dean T. Acheson, Elizabeth W. Twamley, Jared W. Young

**Affiliations:** ^1^Department of Psychiatry, University of California San DiegoLa Jolla, San Diego, CA, USA; ^2^Research Service, San Diego Veteran's Affairs HospitalSan Diego, CA, USA; ^3^VA San Diego Healthcare System, Center of Excellence for Stress and Mental HealthSan Diego, CA, USA

**Keywords:** schizophrenia, cognitive remediation, augmentation, DRD1, nAChR

## Abstract

**Rationale:** Impaired cognitive abilities are a key characteristic of schizophrenia. Although currently approved pharmacological treatments have demonstrated efficacy for positive symptoms, to date no pharmacological treatments successfully reverse cognitive dysfunction in these patients. Cognitively-based interventions such as cognitive remediation (CR) and other psychosocial interventions however, may improve some of the cognitive and functional deficits of schizophrenia. Given that these treatments are time-consuming and labor-intensive, maximizing their effectiveness is a priority. Augmenting psychosocial interventions with pharmacological treatments may be a viable strategy for reducing the impact of cognitive deficits in patients with schizophrenia.

**Objective:** We propose a strategy to develop pharmacological treatments that can enhance the reward-related learning processes underlying successful skill-learning in psychosocial interventions. Specifically, we review clinical and preclinical evidence and paradigms that can be utilized to develop these pharmacological augmentation strategies. Prototypes for this approach include dopamine D1 receptor and α7 nicotinic acetylcholine receptor agonists as attractive targets to specifically enhance reward-related learning during CR.

**Conclusion:** The approach outlined here could be used broadly to develop pharmacological augmentation strategies across a number of cognitive domains underlying successful psychosocial treatment.

## Introduction

The primary treatments for schizophrenia are antipsychotic medications, which target psychotic symptoms, but leave patients with considerable disability due to negative and cognitive symptoms (Harvey and Keefe, 2001; Carter, 2005; Keefe et al., 2007; Mintz and Kopelowicz, 2007). To date, no drugs have been approved for treating negative symptoms or cognitive dysfunction in schizophrenia (Floresco et al., 2005; Geyer, 2010). Although there has evidence for modest antipsychotic-induced improvement in cognition (Bilder et al., 2002; Weiss et al., 2002; Weiner et al., 2004; Keefe et al., 2007), several investigators and clinicians have questioned the real-world clinical relevance of these effects. For example, antipsychotic-induced improvement of patients' ability to recall a 12-word list by a tenth of a word, while statistically significant (Keefe et al., 2007), may not be clinically meaningful (Heinrichs, 2007). Some effect sizes may be greater, but certainly no drug-induced normalization of cognition has occurred for patients with schizophrenia. To address this great unmet therapeutic need (Floresco et al., 2005), the United States National Institute of Mental Health sponsored several initiatives. These initiatives include: (1) Measurement and Treatment Research to Improve Cognition in Schizophrenia (MATRICS; Marder and Fenton, 2004; Marder, 2006); (2) the Treatment Units for Research on Neurocognition in Schizophrenia (TURNS; Buchanan et al., 2007); and (3) Cognitive Neuroscience Treatment to Improve Cognition in Schizophrenia (CNTRICS; Carter and Barch, 2007). The MATRICS group developed a consensus clinical test battery (MATRICS Consensus Cognitive Battery; MCCB) for use in trials of cognitive enhancers considered for Food and Drug Administration approval. TURNS was designed to select and evaluate potential procognitive candidates and CNTRICS is currently developing novel tasks from cognitive neuroscience for use in clinical neuroscience with corresponding animal paradigms. To date, however, there has been limited success in clinical trials of treatments aiming to reverse cognitive deficits in patients with schizophrenia (Javitt et al., 2012; Keefe et al., 2013). A CNTRICS battery may take several years to develop. If testing in rodents occurs first, it may be a decade before a CNTRICS battery will even assess a putative therapeutic drug in humans.

The lack of development of procognitive drugs has impacted investment in the field to the extent that many major pharmaceutical companies are no longer employing researchers in this area, a trend seen in CNS drug-development in general (Nutt and Goodwin, 2011) The failure to develop procognitive drugs for schizophrenia could lie in the fact that nearly all attempts have sought a single treatment for a disease likely resulting from multiple neurodevelopmental insults and subsequent compensatory changes (Bigos et al., 2010; Kleinman et al., 2011). With volumetric and/or morphometric abnormalities in >20 brain regions (Levitt et al., 2010), developing a single treatment to normalize such widespread disturbances seems unlikely. A problem with this level of complexity requires innovative solutions. In this paper, we briefly review the literature on the cognitive remediation (CR) approach to treatment and propose a roadmap for developing pharmaceuticals acting in synergy with CR and other psychosocial treatments aimed at improving cognition in schizophrenia.

## Cognitively-based psychosocial treatments

Partially in response to the limited efficacy of traditional pharmacotherapy on cognitive deficits, multiple approaches to CR have been developed (Wykes et al., 2011). CR is defined as “a behavioral training based intervention that aims to improve cognitive processes (attention, memory, executive function, social cognition, or metacognition) with the goal of durability and generalization” (Wykes et al., 2011). Not meant to replace traditional pharmacotherapy, these approaches attempt to utilize the functional neurocircuitry remaining in patients in order to train them to acquire the capacity, skills, and/or knowledge needed to address a wide range of issues, e.g., cognitive performance, medication adherence, job skills, and community functioning (Swerdlow, 2011; Wykes et al., 2011). Because a variety of techniques exist, these treatments can be individually tailored to focus on deficits specific to a given patient. Such treatments may be a cost-effective adjunct to pharmacotherapy alone (Gutierrez-Recacha et al., 2006).

CR treatments include “restorative” approaches that aim to repair brain functioning and “compensatory” approaches that aim to use strategies to bypass existing deficits (Twamley et al., 2003; Patterson and Leeuwenkamp, 2008; Kern et al., 2009). Restorative treatments typically involve repetitive practice on cognitive tasks in various domains, assuming that such practice will strengthen cognitive performance and generalize to improved functioning in the community. Compensatory approaches attempt to mitigate existing deficits by teaching new skills and cognitive habits (e.g., Twamley et al., 2012). Both approaches use a variety of tools, such as computer-aided tasks, paper-and-pencil tasks, calendars, checklists, and mnemonics, and both involve positive feedback or praise for successful performance of tasks and strategy use. Thus, through positive feedback, participants are encouraged to enhance their skill-set, repeat such performances, and apply their skills in real-life situations.

A growing literature demonstrates the efficacy of CR for improving multiple cognitive and functional domains in schizophrenia patients (Twamley et al., 2003; McGurk et al., 2007; Wykes et al., 2011). A recent meta-analysis of 40 CR studies (Wykes et al., 2011) found moderate effects of CR on cognitive performance (*d* = 0.45) and functioning (*d* = 0.42). Integrated Psychological Therapy, combining elements of CR and other psychosocial therapies, has also been proven effective, with meta-analytic support for effects on cognitive performance (*d* = 0.54), negative symptoms (*d* = 0.41), and social functioning (*d* = 0.41) (Roder et al., 2006).

Other psychosocial treatments for schizophrenia share common features with CR, such as skill learning and positive feedback. Cognitive behavioral therapy (Wykes et al., 2008) focuses on challenging maladaptive thoughts and changing behaviors, whereas social skills training (Kurtz and Mueser, 2008) and social cognitive training (Kurtz and Richardson, 2012) focus on improving social skills and functioning. Although we focus on CR because it targets cognitive functioning most directly, the roadmap for developing pharmacologic augmentations to psychosocial treatments for schizophrenia would apply to other skill-based psychosocial interventions such as these.

As with antipsychotic treatment alone, the success of CR and other psychosocial interventions is limited, with room for improvement remaining. Moreover, psychosocial treatments require considerable clinical resources, as they are typically implemented by highly trained clinicians over months to years, and even so, are not always successful (Kurtz et al., 2007; Dickinson et al., 2010), with mean effect sizes for improvements in the small to medium range. Additionally, these effect sizes have stayed relatively stable across time, despite improving knowledge regarding the nature of cognitive impairment in schizophrenia and treatment delivery technologies (e.g., computer-aided treatments; McGurk et al., 2007). Recently, Swerdlow and colleagues (Swerdlow, 2011; Chou et al., 2012) have called for a focus on identifying pharmacotherapeutics targeting specific components of neurocognition to synergistically augment psychosocial and cognitive treatments such as CR. For instance, utilizing knowledge regarding the neurobiological basis of reinforcement learning or sensory discrimination, pharmacotherapies might facilitate this process within the context of psychosocial therapy. Importantly, using such pharmacotherapies episodically (i.e., treatment given only immediately prior to CR training sessions and discontinued once the course of CR is completed) may eliminate tolerance issues and enable tighter clinical control of medication use. Indeed, a similar strategy is currently being developed in the treatment of anxiety-related disorders, as we discuss below.

## A synergistic pharmaco- and cognitive-intervention approach: lessons from exposure-based psychotherapies

Exposure-based treatments are effective in treating a wide range of trauma and anxiety-related disorders, including obsessive-compulsive disorder, panic disorder, social phobia, and posttraumatic stress disorder (Deacon and Abramowitz, 2004). As with psychosocial treatment for schizophrenia, however, room for improvement exists. For example, only 50–70% of panic disorder patients treated with exposure-based cognitive-behavioral therapy achieve an adequate response following the acute phase of therapy (Furukawa et al., 2006).

Recently, neuroscientists have begun identify the neurobiological and molecular processes mediating the extinction of conditioned fear, the mechanism through which exposure-based therapies are thought to work (Bentz et al., 2010). N-methyl-D-aspartate (NMDA) receptor activity within the basolateral amygdala is an important mediator of fear extinction memory (Davis, 2002). Preclinical animal studies suggested that D-cycloserine (DCS), a partial agonist of the glycine_B_ coagonist site of NMDA receptors (Sheinin et al., 2001), enhanced fear extinction when delivered in a phasic dosing pattern prior to extinction training [see Norberg et al. (2008) for a meta-analytic review]. Clinical research has now documented DCS-facilitation of response to exposure therapy for a range of anxiety disorders (Norberg et al., 2008; De Kleine et al., 2012). In clinical practice, DCS is intended to be delivered approximately 1 h prior to the exposure therapy sessions. DCS is not administered at any other time and is discontinued following the course of exposure therapy. Despite research on DCS enhancement of exposure therapy being in its infancy, clinical research has begun documenting the efficacy of this strategy for a range of anxiety disorders for which exposure is a significant component of psychosocial treatment, including posttraumatic stress disorder, panic disorder, social phobia, obsessive-compulsive disorder, and specific phobia (Norberg et al., 2008; Otto et al., 2010b; De Kleine et al., 2012). In a review of all clinical studies conducted up to 2008, Norberg and colleagues (2008) found an average Cohen's *d* of 0.60 (moderate effect) for DCS augmentation of exposure therapy relative to placebo. Additional compounds with putative augmentative functions, such as oxytocin, valproic acid, and 7,8-dihydroxyflavone, are currently under investigation (Kuriyama et al., 2011; Andero and Ressler, 2012; Acheson et al., 2013).

**Figure 1:**
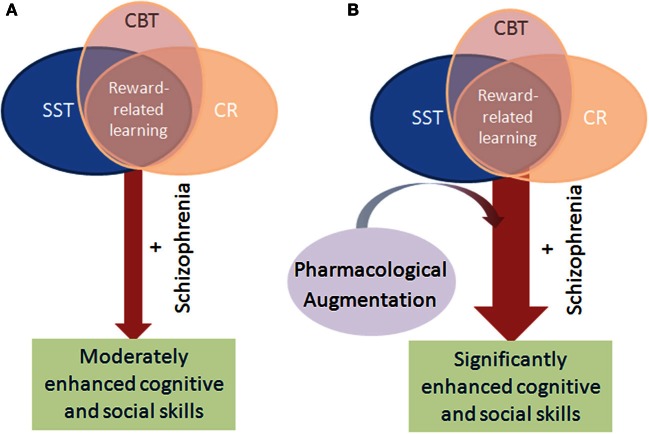
**Schematic of the mechanism by which pharmacological augmentation of psychosocial treatment is proposed to work.** Psychosocial treatment of cognitive deficits in schizophrenia via cognitive remediation (CR), cognitive behavioral therapy (CBT), and social skills training (SST) rely on positive reinforcement to encourage learning in patients. Through such reinforcement learning, the cognitive performance and hence functional outcome of patients with schizophrenia modestly improve over time **(A)**. Patients with schizophrenia exhibit impaired reward-related learning however, which likely negatively impacts the benefit of these psychosocial interventions. By augmenting these interventions with a pharmacological treatment that enhances reward-related learning, it is theorized that patients will gain maximal benefit from the intervention, resulting in greater improvement than with intervention alone **(B)**.

Focus on pharmacological-enhanced neuroplasticity in synergy with psychosocial therapies represents a paradigm shift in the treatment of anxiety-related disorders (Krystal, 2007). Rather than an either-or approach where advances in psychosocial therapy and pharmacotherapy are made independent of one another, this strategy provides a model where knowledge regarding the neurobiological mechanisms underlying the effectiveness of psychosocial therapies is used to identify potential drug targets. This approach is distinct from the traditional model of pharmaco-psychosocial combination treatment (i.e., SSRI and CBT), where each treatment has been developed independently, with independent efficacy, without targeted mechanistic interactive effects (Otto et al., 2010a). The synergistic model has generated success within anxiety disorders because (1) the type of learning thought to mediate effective psychosocial treatment, fear extinction, had been identified, and (2) the neurobiological underpinnings of this type of learning had been fairly well elucidated, enabling the identification of putative drug targets. The development of pharmacological and psychosocial co-therapies for schizophrenia is likely to require a similar breakdown of the type of learning used in CR as well as the neurobiology underpinning that learning. We hypothesize that successful CR may be partially mediated by reward-related learning (Figure 1), and that this type of learning may be ubiquitous amongst the various available iterations of CR. If true, developing a better understanding of reward-related learning in the context of CR would be important for developing pharmacotherapies to augment CR. Below, we present the logic of the importance for reinforcement learning in CR. Moreover, we propose that two major pro-cognitive targets for schizophrenia, the dopamine D1 receptor (DRD1) and the alpha 7 nicotinic acetylcholine receptor (nAChR), may be ideal for augmenting reward-based learning in CR. To reduce patient burden and unnecessary medication use and side-effects, phasic dosing of cognitive-enhancing agents could be given only in conjunction with CR, as these drugs are unlikely to produce cognitive benefit when not paired with CR learning activities.

## Preclinical research for pharmacological augmentation of CR

### Reward-based learning in CR

Using animal research in developing pharmaceutical augmentations for CR is hampered by the fact that CR is not conducted in animals. Such was also the case for augmenting exposure-based therapies however, but by identifying the primary mechanism of learning, i.e., fear extinction, it was possible to research this mechanism and identify putative treatments, e.g., DCS. Thus, to aid research for augmenting CR, it is important to identify a sufficiently narrow mechanism—thought to at least partially underlie the effectiveness of psychosocial treatments—which can be pharmacologically targeted. Unfortunately, little is currently known about the mechanism of action underlying CR. Most current research examines cognitive performance or community functioning as primary dependent variables, rather than changes at a molecular level necessary for drug discovery (Twamley et al., 2003; Pfammatter et al., 2006; McGurk et al., 2007; Wykes et al., 2011).

Positive feedback from facilitators, peers, or computers is used in CR to increase desired behaviors as participants learn new skills. Although these are features of almost all CR interventions, two specific examples of the use of positive reinforcement in CR are Silverstein and colleagues' “attention shaping” program (Silverstein et al., 2009) and Vinogradov and colleagues' (Fisher et al., 2010) computer-delivered neuroplasticity-based treatment program to enhance auditory sensory discrimination and verbal learning. In the attention-shaping program, individual attentiveness goals are set for each patient, and the patient is reinforced both verbally and with a token when the goal is met. In the neuroplasticity-based treatment program, correct responses on a verbal memory task are consistently followed by rewarding stimuli to strengthen learning and thereby future verbal memory performance. Both programs have shown promising results on their target cognitive construct, attention and verbal memory respectively (Silverstein et al., 2009; Fisher et al., 2010). Vinogradov and colleagues (2009b) have shown that patients with a heavier anticholinergic burden have a poorer response to CR, and that serum brain derived neurotrophic factor increases after CR (Vinogradov et al., 2009a).

Patients with schizophrenia exhibit deficits in reward-related learning (Waltz et al., 2007, 2011; Weiler et al., 2009), perhaps associated with reduced brain activation following reward-predicting stimuli in unmedicated patients (Juckel et al., 2006). This deficit probably reflects a fundamental change due to the disease process and not a medication-induced change. Although poor reward-related learning may also reflect lower motivational levels in these patients (Keefe et al., 2011), it is not associated with any negative symptoms and hence unlikely to simply reflect a reduction in reward value (Waltz et al., 2007, 2011; Strauss et al., 2011). Given the positive reinforcement used in CR and other psychosocial treatments, impaired reinforcement learning in patients with schizophrenia may slow treatment progress (Weiler et al., 2009; Strauss et al., 2011).

Pharmacological augmentation of reward-based learning has the potential to improve psychosocial treatments for schizophrenia. In the clinic, pharmaceutical compounds that have been shown to influence the strength and rate of reward learning could be delivered prior to sessions of controlled CR training. Influencing the strength of initial learning might allow for more comprehensive skill acquisition, as well as increased durability of treatment effects over time. Increasing the speed of learning could help to make these treatments more cost-effective and appealing to both providers and service users. For instance, Vinogradov and colleagues (Fisher et al., 2010) reported the most gain from their neuroplasticity-based verbal memory treatment in patients who underwent at least 100 h of training. Pharmacological augmentation might reduce the training hours required for clinically significant gains, such as has been accomplished with exposure-based psychotherapies for anxiety (Norberg et al., 2008). In order to achieve this goal, there is a clear need for basic preclinical research demonstrating the potential to influence reward-based learning. Preclinical research can also be used to reveal mechanisms underlying reward-based learning as well as testing putative pharmacotherapeutics.

## Preclinical strategies for developing treatment options to augment CR

Just as developing augmentations to exposure-based therapies required an understanding of fear extinction, the use of positive reinforcement in CR supports the need to understand reward-related learning mechanisms. It is clear that learning via positive feedback has a different underlying mechanism from learning via punishment or negative reinforcement. To identify mechanisms underlying this learning, tasks with cross-species translational relevance across species should be utilized. Although there are numerous reward-related tasks that require training the animal to perform them, not all have a suitable throughput or cross-species relevance. For example, training rodents to perform the 5-choice serial reaction-time task can take more than 2 months (Lustig et al., 2012), during which time repeated treatment effects would be impractical. Moreover, most laboratory tests of learning in healthy humans occur within a single test session. By using tasks with cross-species translational validity for these aspects of learning (Ragland et al., 2009; Young et al., 2009, 2012b), positive treatment findings in normal animals could be confirmed in healthy humans (Ragland et al., 2009) prior to testing in patients.

Thus, the use of positive reinforcement in CR supports the need to focus animal research on reward-related learning mechanisms. Theoretically, pharmacologically-enhanced learning of this type that can be observed both in healthy controls and patients with schizophrenia would be useful because this would suggest a similarly intact neural mechanism that can be engaged in patients (Swerdlow, 2011). Two primary targets identified from MATRICS for the development of procognitive compounds, the DRD1s and α7 nAChRs (Tamminga, 2006; www.matrics.ucla.edu), have been implicated in reward-related learning. These targets represent logical starting points to exemplify developing reward learning-enhancing pharmaceuticals.

### DRD1 and reward-related learning

Evidence supports dopamine-mediated reward-related learning whereby environmental stimuli and action sequences are encoded via spikes in dopamine levels (Schultz, 2002; Montague et al., 2004; Tobler et al., 2005). Dopamine firing rates may also underlie aversive-related learning, but at lower and shorter durations compared with rewarding stimuli (Joshua et al., 2008; Matsumoto and Hikosaka, 2009; Morris et al., 2010; Glimcher, 2011), supporting the role of dopamine in the reward-prediction-error hypothesis (Glimcher, 2011).

The reward-prediction-error hypothesis provides a putative mechanism underlying Thorndike's Law of Effect. Dopamine fires in response to an unpredicted reward, but once predictable, dopamine firing ceases (Enomoto et al., 2011; Glimcher, 2011). Thus, dopamine strengthens the synaptic connection between reward and action. Importantly, schizophrenia patients exhibit reduced prediction-error signals compared with controls (Gradin et al., 2011). Moreover, striatal dopaminergic neurons are involved in long-term potentiation (LTP), thought to underlie learning (Surmeier et al., 2009), which is also altered in schizophrenia patients (Hasan et al., 2011).

Striatal DRD1s are linked to the direct pathway that stimulates the thalamus and cortex (Gerfen and Engber, 1992; Morris et al., 2010). DRD1 stimulation likely strengthens synaptic connections, promoting LTP (Wolf et al., 2004). Similarly, DRD1 knockout mice exhibit altered LTP and impaired associative learning (Matthies et al., 1997; Granado et al., 2008; Ortiz et al., 2010).

Evidence for DRD1 agonist-induced enhancement of reward-related learning is limited, however, in part because few full agonists are available—most studies utilize partial agonists (e.g., SKF38393; Zhang et al., 2008). Testing whether full DRD1 agonists (e.g., doxanthrine; Przybyla et al., 2009; McCorvy et al., 2012) can improve reward-related learning in animals will be an important next step (Young et al., 2010). Other compounds, such as amphetamine or modafinil, may exert their reward-related effects on behavior in part via a dopamine D1 receptor mechanism (Qu et al., 2008; Young, 2009; Young and Geyer, 2010; Liu et al., 2011; Scoriels et al., 2013). Although there are other diverse mechanisms of actions (Minzenberg and Carter, 2008; Scoriels et al., 2013) of these treatments, it would be useful to determine whether these treatments may be useful to augment CR, and if their pro-learning effects are indeed mediated by the dopamine D1 receptor. Determining treatments with greater dopamine D1 selectivity of effects may be important given the possible role dopamine D2 receptors play in the blockade of learning. Because all antipsychotics are dopamine D2 antagonists, determining the interactive effects of a D1 receptor agonist with D2 receptor blockade will also be important (Young et al., 2012a). The effects of dopamine D2 blockade on rodent cognition alone and in models of schizophrenia have been discussed elsewhere (Hagan and Jones, 2005; Young et al., 2009, 2012c). Moreover, the positive and negative effects of dopamine D2 receptor blockade on cognition in schizophrenia have also been discussed (Harvey and Keefe, 2001; Buchanan et al., 2005; Keefe et al., 2007, 2011). In terms of augmenting CR with a DRD1 agonist or any other treatment, it will be important to identify that any positive treatment will remain so in the presence of chronic antipsychotic treatment (Floresco et al., 2005).

After MATRICS identified the dopamine D1 receptor as a primary target for cognition enhancement in schizophrenia, the NIMH-funded TURNS initiative have assessed the efficacy of the full dopamine D1 agonist dihydrexidine (Mu et al., 2007). Very low doses have had to be used to date however, given orthostatic hypertension, hypotension, and tachycardia that have occurred with intravenous administration (Blanchet et al., 1998), and while it has been tolerated with a subcutaneous dose in patients with schizophrenia it is unclear whether such complications arose (George et al., 2007). Phasic dosing during CR may be an ideal format for administering a treatment with such side-effects (see below).

### α7 nAchRs and reward-related learning

Nicotine (the prototypical ligand of nAChRs) enhances learning in healthy humans, schizophrenia patients, and animals (Levin et al., 1998; Newhouse et al., 2004; Poltavski and Petros, 2006; Barr et al., 2008; Myers et al., 2008; D'Souza and Markou, 2012). Acute mecamylamine (a nAChR antagonist) impairs learning (Newhouse et al., 1992), while ABT-418 (an α4β2 nA ChR agonist) improved learning in Alzheimer's disease patients (Potter et al., 1999). Schizophrenia patients exhibit higher smoking rates, which may be a form of self-medication (Kumari and Postma, 2005). Pathological abnormalities of α7 nAChRs in schizophrenia patients (e.g., 15q13-15) have been linked to poor sensory gating (Freedman et al., 1997). Lower α 7 nAChR protein levels are observed in the post-mortem brains of patients with schizophrenia and are associated with cognitive dysfunction (Martin-Ruiz et al., 2003). Moreover, α7 nAChR mRNA expression may be regulated by neuregulin-1 genetic variation (Mathew et al., 2007), a genetic risk factor for schizophrenia (Stefansson et al., 2002, 2003; Harrison and Law, 2006; Law et al., 2006). The α 7 nAChR modulates numerous mechanisms throughout the brain that are relevant to schizophrenia and its pathophysiology (Bencherif et al., 2012) and may also modulate aspects of cognition (Thomsen et al., 2010). In line with such proposals, α 7 nAChR knockout mice exhibit impaired reward-related learning across numerous paradigms (Young et al., 2004, 2007a, 2011; Keller et al., 2005; Levin et al., 2009) and impaired LTP (Dziewczapolski et al., 2009), whereas α 7 nAChR agonists enhance LTP (Lagostena et al., 2008; Kroker et al., 2011). Finally, aversive-motivated learning is intact in α 7 nAChR knockout mice (Paylor et al., 1998), suggesting that this receptor specifically contributes to reward-related learning. Thus, α 7 nAChR abnormalities may impact reward-learning in schizophrenia patients, representing a potential pharmacotherapeutic target, especially as an agonist for this receptor would be less likely than nicotine to be addictive (Martin et al., 2004; Levin and Rezvani, 2006).

Early clinical tests of a partial α 7 nAChR agonist (DMXBA) were promising with regard to improving cognition in patients with schizophrenia (Olincy et al., 2006), but these effects were not replicated in larger studies (Freedman et al., 2008). When given to rats, daily DMXBA- or nicotine-injections improved learning in aged rats (Levin et al., 1990; Arendash et al., 1995a,b; Taylor et al., 2005). Few studies have characterized the effects of such agonists specifically on reward-related learning, however. In a recent review, Hahn and colleagues (2013) discuss the merits of targeting nAChRs as putative add-ons to CR. Primarily, the authors emphasized the ability of nAChR agonists to modestly improve several aspects of cognition, such as attention (Grottick et al., 2003; Young et al., 2004, 2013; Rezvani et al., 2009; Hahn et al., 2011) and working memory (Levin, 2002; Levin et al., 2002; Young et al., 2007b; Rushforth et al., 2010). Thus, they propose that assessing the efficacy of targeted nAChR treatment with CR may provide the best opportunity to enhance cognition and hence functioning in patients with schizophrenia. Recently, Lieberman et al. (2013) demonstrated that the full α7 nAChR agonist TC-5619 can modestly improve cognition in patients with schizophrenia. Importantly, this improvement was not blocked by current smoking, an important aspect if such a treatment were used in combination with CR.

α7 nAChR agonists should be assessed in combination with CR. Treatment with a cognition-enhancer without structured CR could enhance aspects of learning and habits not conducive to recovery (e.g., paranoid associations). Further, the extant studies on α 7 nAChR agonists have used a chronic dosing strategy, rather than applying phasic dosing in conjunction with targeted CR. Such phasic dosing may also be more useful in patients that smoke or are ex-smokers, given the high rate of smoking in schizophrenia (Dolan et al., 2004; Dwoskin et al., 2009; D'Souza and Markou, 2012).

## Methods for assessing reward-related learning and its components in rodents

There are myriad methods for assessing reward-related learning in rodents. In order to specifically identify treatments that cross the species gap, however, focus should be placed on tasks that can be assessed in rodents and humans (Floresco et al., 2005; Young et al., 2009). Given that exposure-based therapy is essentially similar to what is described as fear extinction in rodents—leading to the development of DCS to augment this mechanism (Norberg et al., 2008)—a reasonable starting point would be to use similar learning paradigms across species.

The CNTRICS initiative identified tasks that can assess reinforcement learning in humans, with putative tasks for testing across species (Ragland et al., 2009). These tasks include the transitive inference paradigm (TIP) and the probabilistic selective task. Given that the task exists in both species, the TIP has evidence for cross-species translational validity (Young et al., 2012b). For example, bilateral hippocampal-lesioned mice exhibit a similar pattern of deficits as do humans with hippocampal lesions (Dusek and Eichenbaum, 1997) and share some similarities to patients with schizophrenia (Titone et al., 2004). The TIP deficits of patients with schizophrenia are also similar to mice with bilateral lesions of the prefrontal cortex, however, where inference deficits are observed but no improvement of extreme value stimuli are observed (Devito et al., 2010). Importantly, TIP deficits are also observed in human subjects with frontal lobe damage, although they can make first order associations readily (Waltz et al., 1999). The probabilistic selective task is also available in both humans and rodents. Although only two studies have been conducted in rats and mice (Bari et al., 2010; Amodeo et al., 2012), there has been pharmacological consistency between the two rodent species as well as tests in humans (Chamberlain et al., 2006). Importantly, this probabilistic reward task can also differentiate between model-based (learning best strategy) from model-free (repeat responding of rewarded actions) learning (Daw et al., 2011). Such model-based learning is important because there are numerous aspects during CR that must be assimilated into an overall model.

There may of course be other aspects of cognition that contribute to learning from CR, e.g., attention and working memory. As with reinforcement learning, there are tasks that are available to measure these cognitive domains across species. These tasks have been reviewed in detail elsewhere (Young et al., 2009; Dudchenko et al., 2012; Lustig et al., 2012). Importantly, any pharmacological enhancement of any of these tasks in normal rodents could be validated in healthy humans. If the treatment proved modestly efficacious across species, the same treatment could be tested in people with schizophrenia to ensure similar efficacy prior to being tested during CR.

## Conclusions and future directions

As described above, pharmaceutical companies have reduced their investment in pro-cognitive drugs due in part to a lack of positive pro-cognitive effects of treatments for schizophrenia (Nutt and Goodwin, 2011). The approach proposed here provides an avenue by which the pharmaceutical industry can reinitiate their role in developing such treatments. Given that there is another avenue by which treatments—that may have had limited efficacy in the MCCB—could enhance cognition in schizophrenia, clinical trials could be redesigned as described here and elsewhere (Swerdlow, 2011; Chou et al., 2012). Such clinical trials would require phasic treatment only just prior to CR training, as is conducted in DCS treatment combined with behavioral treatment for anxiety disorders. There are several benefits to phasic administration of treatments during CR; (1) Dosing can be controlled by the clinician; (2) Treatments with short half-lives can be used; (3) Side-effects resulting from treatment can be better controlled for (e.g., dihydrexidine); and (4) The treatment is only required for as long as the CR occurs.

**Figure 2:**
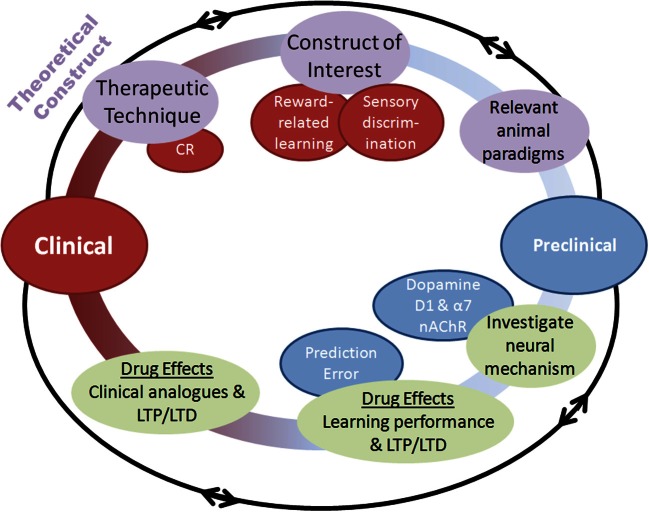
**Schematic of the process by which pharmacological augmentations to psychosocial treatments may be developed.** Both clinical and preclinical phases are represented, with overlapping and multidirectional processes. Clinical. (1) A therapeutic technique of interest is identified such as cognitive remediation (CR), (2) An underlying cognitive construct to be targeted for augmentation is defined, (3) Animal paradigms with the ability to assay this construct are identified or created. Preclinical. (4) Neural mechanisms underlying the construct of interest are identified, (5) Pharmacological compounds interacting with those neural mechanisms are assessed in animal paradigms for their ability to augment performance, (6) Putative pharmacological augmentations are tested for effectiveness using human clinical analogues in the laboratory before randomized clinical testing. Importantly, this process highlights the bi-directional application of research at each stage, with theoretical constructs leading to testing of hypotheses in animals, which can further refine these constructs.

This paradigm-shift of combining psychosocial treatment with pharmacotherapies that enhance the underlying neural mechanisms of psychosocial treatments could be essential in enhancing cognition in schizophrenia (Swerdlow, 2011). Designing treatments that work synergistically with CR is essential. Reward-related learning appears a reasonable starting point for this strategy. This strategy requires: (1) Confirming the contribution of reward-related learning toward positive CR effects, (2) Elucidating the underlying reward-related learning mechanisms supporting CR; and (3) Identifying treatments that can augment the neurobiological processes supporting these mechanisms (Figure 2). Biomarkers of treatment effects on reward learning, such as LTP (Hasan et al., 2011), or reward-prediction signals (Gradin et al., 2011), can improve the chance of drug-development translating across species (Luck et al., 2011). This strategy may have broad utility across a number of different cognitive domains in addition to reward learning. Further, other disorders characterized by cognitive dysfunction outside of schizophrenia (e.g., bipolar disorder and autism) may also benefit from this approach.

Putative treatments such as DRD1 and α 7 nAChR agonists may be more effective when used phasically to augment CR as compared to a chronic dosing strategy alone. More general treatments already available, such as modafinil or available nAChR agonists, could be used initially for proof-of-concept. Other targets could be gained from understanding mechanisms underlying neuroplasticity [e.g., LTP, see review by Nicoll and Roche (2013)]. Alternatively, identifying the molecular, structural, and functional correlates of environmental enrichment-dependent plasticity (McOmish and Hannan, 2007) may provide other targets for developing augmentation treatments. Ultimately, combining psychosocial and pharmacological treatments for schizophrenia may be the best opportunity to improve functional outcomes for these patients. We hope that this discussion stimulates research in the field toward that end.

### Conflict of interest statement

The authors declare that the research was conducted in the absence of any commercial or financial relationships that could be construed as a potential conflict of interest.
